# An Attempt to Develop a New Treatment Strategy for Rare Refractory Gynecological Malignancies: The Japanese Gynecologic Oncology Group

**DOI:** 10.31662/jmaj.2023-0024

**Published:** 2023-09-29

**Authors:** Shogo Shigeta, Muneaki Shimada, Shiro Suzuki, Hiroaki Kajiyama, Katsutoshi Oda, Kazuhiro Takehara, Masaki Mandai, Daisuke Aoki, Takayuki Enomoto, Aikou Okamoto

**Affiliations:** 1 Department of Obstetrics and Gynecology, Tohoku University School of Medicine, Miyagi, Japan; 2 Department of Clinical Biobank, Advanced Research Center for Innovations in Next-Generation Medicine, Tohoku University, Miyagi, Japan; 3 Department of Gynecologic Oncology, Aichi Cancer Center Hospital, Aichi, Japan; 4 Department of Obstetrics and Gynecology, Nagoya University Graduate School of Medicine, Aichi, Japan; 5 Division of Integrative Genomics, Graduate School of Medicine, The University of Tokyo, Tokyo, Japan; 6 Department of Gynecologic Oncology, National Hospital Organization Shikoku Cancer Center, Ehime, Japan; 7 Department of Gynecology and Obstetrics, Graduate School of Medicine, Kyoto University, Kyoto, Japan; 8 Department of Obstetrics and Gynecology, Keio University School of Medicine, Tokyo, Japan; 9 Department of Obstetrics and Gynecology, Niigata University Graduate School of Medical and Dental Sciences, Niigata, Japan; 10 Department of Obstetrics and Gynecology, Jikei University School of Medicine, Tokyo, Japan

**Keywords:** Gynecological malignancies, Rare cancer, Ovarian clear cell carcinoma, Clinical trials

## Abstract

Platinum-based combination chemotherapy has been a frontline therapeutic strategy for advanced ovarian cancer. Although patients with ovarian high-grade serous carcinoma (HGSC) respond well to the combination therapy, those with relatively rare histologic subtypes, such as mucinous or clear cell carcinoma of the ovary (OCCC), show resistance to platinum-based chemotherapy. Even with the recently developed maintenance therapies using molecular targeted inhibitors for ovarian cancers, such as bevacizumab or poly (ADP-ribose) polymerase (PARP) inhibitors, the prognosis of non-HGSC ovarian cancers is unsatisfactory. To overcome the limitations in the treatment of rare ovarian cancers, the Japanese Gynecologic Oncology Group (JGOG) has launched a comprehensive project utilizing publicly available genomic databases, including a national clinico-genomic database maintained by the Center for Cancer Genomics and Advanced Therapeutics (C-CAT). JGOG, a leading group in Japan that conducts clinical trials for the treatment of gynecological malignancies, also established a nationwide network through the long-standing efforts of all participants. Currently, JGOG is engaged in a phase II international clinical trial (CYH33-G201: jRCT2031210216), targeting OCCC with *PIK3CA* hotspot mutations. The CYH33-G201 trial is sponsor-initiated, and JGOG, in collaboration with pharmaceutical companies, is actively recruiting participants. To expand the functions of the nationwide network that JGOG had already established, we held explanatory meetings for this clinical trial in nine different areas throughout Japan to promote the penetration of the CYH33-G201 trial. Through C-CAT database analysis, we estimated that approximately 40% of the patients with OCCC harbored at least 1 of the 17 *PIK3CA* hotspot mutations designated in the CYH33-G201 trial. JGOG will continue the challenge of establishing novel treatment strategies for rare refractory cancers that will benefit patients suffering from gynecological malignancies, especially those who do not receive satisfactory standard treatment and care.

## History of the Development of Therapeutic Strategies for Gynecological Malignancies

Recent advances in molecular biology have led to the development of sophisticated therapies for malignant tumors. Cisplatin was approved for cancer therapy in Japan in 1983, and multidisciplinary methods, including cytoreductive surgery, were established for the treatment of advanced epithelial ovarian cancer (EOC), an intractable gynecological cancer. The results of a randomized phase III trial (GOG111) published in 1996 prompted the use of combination chemotherapy with paclitaxel and cisplatin (TP therapy) as the standard chemotherapy for advanced EOC, thereby improving the prognosis for patients with advanced EOC ^(1)^. Later, the findings of the GOG158 trial highlighted the noninferiority and lower toxicity of the paclitaxel and carboplatin (TC) therapy compared to TP therapy. Since then, TC therapy has been considered a frontline therapy. Although many cytotoxic anticancer chemotherapeutic agents were subsequently tested to further improve the prognosis of advanced EOC in international randomized phase III trials, known as mega-trials, none of these chemotherapeutic regimens was demonstrated to be superior to TC therapy. However, the results of many retrospective studies have revealed that ovarian clear cell carcinoma (OCCC) and ovarian mucinous carcinoma (OMC) are resistant to TC therapy, highlighting the importance of developing therapeutic strategies focused on these histological subtypes. Although cases of OCCC and OMC are less common than those of high-grade serous carcinoma (the most common histological subtype of EOC) and the proportion of advanced OCCC and OMC cases is low, the prognosis for advanced or recurrent cases of OCCC and OMC is extremely poor. Overall, the development of treatment strategies for these rare refractory cancers is a very important issue.

The results of some landmark international trials demonstrated the benefit of the angiogenesis inhibitor bevacizumab in combination with TC therapy or as a maintenance therapy for advanced or recurrent EOC ^(2)^. Subsequently, the efficacy of maintenance therapy using poly (ADP-ribose) polymerase (PARP) inhibitors such as olaparib and niraparib was demonstrated, leading to the speculation that a dramatic improvement in the prognosis of advanced or recurrent EOC could be achieved ^(3)^. Unfortunately, Japanese institutions have not been able to fully participate in international collaborative trials and rely heavily on the results of overseas clinical trials for the development of new therapies. Thus, there is not enough information available on the efficacy and the safety of new drugs for the Japanese population.

## Japanese Gynecologic Oncology Group

The Japanese Gynecologic Oncology Group (JGOG) is a collaboration of major university hospitals and cancer centers in Japan. JGOG originates from a cervical cancer chemotherapy research group founded in 1981, which later developed into a gynecological chemotherapy research group. In 2002, the group received a nonprofit organization certificate as JGOG. The mission of JGOG is to establish optimal and up-to-date treatment strategies for patients with gynecological malignancies. JGOG has been the largest clinical trial group since its establishment in Japan. As of 2022, JGOG includes 181 registered participating facilities and 1,046 active members.

JGOG has completed or participated in numerous important clinical trials, including randomized clinical trials (RCTs). For example, some of the JGOG facilities participated in the aforementioned GOG218 trial, which demonstrated the benefit of using bevacizumab in ovarian cancer ^(2)^. This led to the approval of government insurance coverage for a molecular targeted therapy for gynecological malignancies for the first time in November 2013 in Japan. In addition, JGOG completed a global phase III trial (JGOG3017), in which irinotecan plus cisplatin therapy was compared with TC therapy for advanced OCCC, with a focus on the development of treatment methods according to histological subtypes ^(4)^. Recently, Japanese institutions associated with JGOG have participated in international phase III trials, which are being conducted mainly by the Gynecologic Cancer Intergroup (GCIG), to evaluate the usefulness of molecular targeted therapies. The completed RCTs and ongoing trials led by JGOG at the time of submission are summarized in Table 1.

**Table 1.** Completed and Ongoing Clinical Trials Led by JGOG.

Title		JGOG ID	Target Disease	Identifier
Completed randomized clinical trials	Randomized phase III trial of AP (doxorubicin + cisplatin) therapy, DP (docetaxel + cisplatin) therapy, and TC (paclitaxel + carboplatin) therapy as adjuvant chemotherapy for endometrial cancer at a high risk of recurrence	JGOG2043	Endometrial Cancer	UMIN000000522
	Randomized phase III trial of conventional paclitaxel and carboplatin versus dose dense weekly paclitaxel and carboplatin in patients with newly diagnosed stage II-IV Mullerian carcinoma (ovarian epithelial, primary peritoneal, or fallopian tube cancer)	JGOG3016	Ovarian Cancer	C000000183
	Randomized phase III trial of paclitaxel plus carboplatin (TC) therapy versus irinotecan plus cisplatin (CPT-P) therapy as a first line chemotherapy for clear cell carcinoma of the ovary	GCIG/JGOG3017	Ovarian Cancer	UMIN000000499
	A randomized phase II/III trial of intravenous (IV) paclitaxel weekly plus IV carboplatin once every 3 weeks versus IV paclitaxel weekly plus intraperitoneal (IP) carboplatin once every 3 weeks in women with epithelial ovarian, fallopian tube or primary peritoneal cancer	GOTIC/JGOG3019	Ovarian Cancer	UMIN000003670/NCT01506856
Ongoing trials/studies	Adjuvant chemotherapy versus radiotherapy for postoperative cervical cancer; a phase III trial	JGOG1082	Cervical Cancer	jRCTs041190042
	Clinicopathological study on glassy cell carcinoma of the cervix	JGOG1086S	Cervical Cancer	UMIN000050237
	A non-randomized confirmatory trial of minimum invasive laparoscopic radical hysterectomy (new-Japanese LRH) for patients with early-stage cervical cancer	JGOG1087	Cervical Cancer	UMIN000045224
	Phase II trial of repeated high dose luteal hormone therapy for intrauterine recurrence following fertility preserving therapy for atypical endometrial hyperplasia or endometrial cancer	JGOG2051	Endometrial Cancer	jRCT031200256
	Phase II study of niraparib in recurrent or persistent rare fraction of gynecologic malignancies with homologous recombination deficiency	JGOG2052	Uterine Leiomyosarcoma and Others	jRCT2031210264
	Phase III trial of stage I ovarian cancer after surgery	JGOG3020	Ovarian Cancer	jRCTs031180423/NCT04063527
	Prospective cohort study of germline variant carriers with BRCA1 or BRCA2	JGOG3024	Ovarian Cancer	UMIN000028740/NCT03296826
	Assessment of the efficacy and safety of pembrolizumab for ovarian squamous cell carcinoma	JGOG3029	Ovarian Cancer	jRCT2031220701
	An observational study to investigate the safety and efficacy of olaparib combined with bevacizumab therapy after primary chemotherapy plus bevacizumab for Japanese patients with advanced ovarian, fallopian tube, or primary peritoneal cancer	JGOG3030	Ovarian Cancer	UMIN000047107
	An observational study to investigate the safety and efficacy of niraparib for Japanese patients with recurrent ovarian cancer: maintenance therapy for platinum-sensitive recurrent treatment and monotherapy for late-line treatment	JGOG3031	Ovarian Cancer	UMIN000046970

## Utilization of Comprehensive Genomic Profiling (CGP) and the Center for Cancer Genomics and Advanced Therapeutics (C-CAT) Database

In Japan, CGP is available for patients with solid malignant tumors without an established standard treatment or those with advanced malignant diseases who have completed or who are expected to complete the standard treatment. The expenses of CGP have been covered by the public insurance scheme since June 2019. In parallel, the Center for Cancer Genomics and Advanced Therapeutics (C-CAT) was established in June 2018. C-CAT aggregates genomic and clinical information from various cancer gene panel tests and delivers a report of the results of its own analysis (C-CAT survey results). The genomic and clinical information gathered by C-CAT is a valuable resource, which has the potential to benefit cancer care in many ways, including drug development in Japan, promotion of new clinical trials, prediction of risk of developing side effects, and improvement of panel test performance. Currently, academic researchers at government-designated cancer genomic medicine hospitals can use C-CAT databases for research use under the approval of both their institutional ethics committee and the C-CAT review board. For developing new treatment strategies for rare refractory gynecological cancers such as OCCC, JGOG is now actively referring to the genome analysis data, including the C-CAT database, an example of which is described next.

## CYH33-G201 Trial in Association with the Patient Recruitment System Organized by JGOG

The CYH33-G201 trial (jRCT2031210216, NCT05043922) is a phase II, open-label, multicenter study that is being conducted to evaluate the efficacy and safety of CYH33 (a selective PI3Kα inhibitor) monotherapy in patients with recurrent or persistent OCCC who harbor at least 1 of the 17 designated hotspot mutations in *PIK3CA* (institutional review board [IRB] number: #T4962). The planned target accrual is 86 globally and 36 in Japan. A targeted genome screening is available as a prescreening test to confirm the presence of these specific genetic mutations for the CYH33-G201 trial. However, only 10 of the 17 designated genetic mutations are detectable in the prescreening test. Thus, CGP has an advantage for genomic screening because all 17 of the designated mutations in *PIK3CA* are detectable. In addition, information regarding the frequency of each of the 17 mutations is very helpful in estimating the number of patients for the accrual of genome-matched clinical trials. Under the approval of both the institutional ethics committee (#2021341G) and the C-CAT review board (#CDU2022-026N), we analyzed *PIK3CA* mutations in OCCC. Importantly, 149 (58.4%) of the 255 patients with OCCC registered in the C-CAT database were positive for *PIK3CA* mutations, and 104 (40.8%) had hotspot mutations that were listed in the eligibility criteria for inclusion in the CYH33-G201 trial.

**Figure 1.**
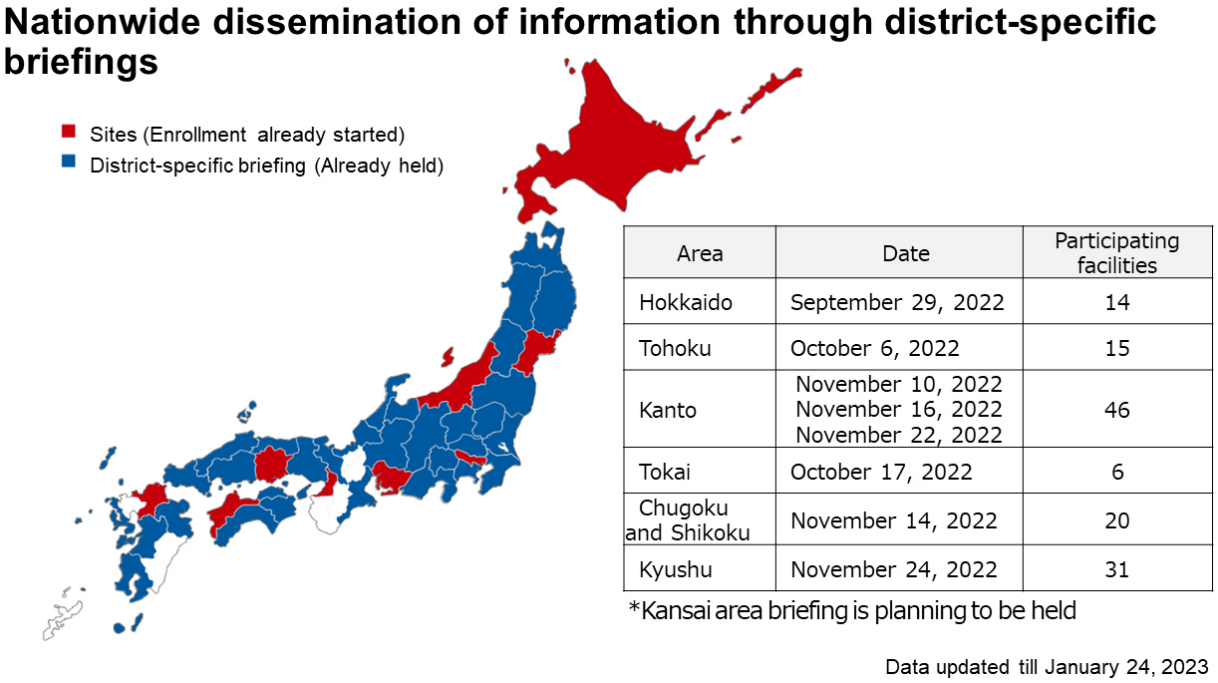
Area map demonstrating the progress of patient enrollment and district-specific briefing. Areas where patient enrollment has already started and where district-specific briefing has been held are shown in red and blue, respectively.

Although cases of OCCC occur more frequently in Japan than in Europe and the United States, it is difficult to complete the enrollment of the CYH33-G201 trial within only high-volume centers in Japan because of its rarity. Therefore, we consider that it is necessary to establish a new system to recruit patients with rare gynecological malignancies such as OCCC from nationwide facilities regardless of center volume. JGOG has established a patient referral system to facilitate the registration of patients to be included in international collaborative trials, but the system has not functioned well in practice. On receiving the introduction of the CYH33-G201 trial, the patient referral system has been optimized and is currently being fully utilized to encourage patients with advanced or recurrent OCCC at each JGOG facility to consider the CYH33-G201 trial as one of the therapeutic options. To promote enrollment, web meetings have been held in nine areas across the country (Figure 1). In addition, a document outlining the salient points of this study was mailed to the JGOG facilities in each area to publicize this study as well as the JGOG referral system. As a result, 26 patients were included as of May 2023 in the CYH33-G201 trial, which began in late August 2022.

## Conclusions and Future Vision of JGOG for Rare Gynecological Malignancies

JGOG has begun an investigator-initiated clinical trial (JGOG2052: jRCT2031210264) to develop a new treatment strategy for rare refractory gynecological malignancies with homologous recombination deficiency, including uterine leiomyosarcoma (IRB number: #I021-002) ^(5)^. A phase II study (JGOG3029: jRCT2031220701, NCT05737199) is also planned to examine the efficiency of an immune checkpoint inhibitor for patients with ovarian squamous cell carcinoma (IRB number: #I022-002). JGOG will expand the system established in the CYH33-G201 trial for the rapid and efficient promotion of the development of treatment strategies for rare refractory gynecological malignancies. In this way, researchers involved with JGOG want to demonstrate the quick, accurate, and safe development of new treatment strategies to the global community. Moreover, JGOG encourages international collaborative trials for devising new treatment strategies and intends to provide useful therapeutic options to women suffering from rare refractory gynecological malignancies.

## Additional Remarks

Each center independently obtained approval from their IRB, and written consent was obtained from all patients to publish the information regarding the clinical trials mentioned in the manuscript. The approval number issued by the IRB at the representative center for each trial is shown in the main text.

## Article Information

### Conflicts of Interest

K.O. received research funds from Konica Minolta, Inc., and lecture fees from Chugai Pharmaceutical Co., Ltd.; D.A. received honoraria from AstraZeneca K.K., Chugai Pharmaceutical Co., Ltd., Takeda Pharmaceutical Company, Ltd., MSD K.K., Eisai Co., Ltd., Genmab K.K., and Myriad Genetics, Inc.; A.O. received research funds from Meiji Holdings Co., Ltd., Taiho Pharmaceutical Co., Ltd., Chugai Pharmaceutical Co., Ltd., ASKA Pharmaceutical Co., Ltd., Mochida Pharmaceutical Co., Ltd., MSD K.K., Eisai Co., Ltd., Takeda Pharmaceutical Company, Ltd., Linical Co., Ltd., Pfizer Japan Inc., Gyne Mom Co., Ltd., Terumo Corporation, Kissei Pharmaceutical Co., Ltd., AstraZeneca K.K., Tsumura Co., Daiichi Sankyo Co., Ltd., Fuji Pharma Co., Ltd., and Nippon Shinyaku Co., Ltd.; A.O. received honoraria from Takeda Pharmaceutical Company, Ltd., AstraZeneca K.K., MSD K.K., Mochida Pharmaceutical Co., Ltd., Bayer Holding Ltd., Kaken Pharmaceutical Co., Ltd., ASKA Pharmaceutical Co., Ltd., Chugai Pharmaceutical Co., Ltd., Kissei Pharmaceutical Co., Ltd., Fuji Pharma Co., Ltd., Zeria Pharmaceutical Co., Ltd., and Eisai Co., Ltd.; A.O. received support for attending meeting from AstraZeneca K.K., Johnson & Johnson K.K., and Takeda Pharmaceutical Company, Ltd.

### Acknowledgement

The authors would like to thank Editage (www.editage.com) for English language editing.

### Author Contributions

Conceptualization: M.S., H.K., K.O., K.T., M.M., D.A., T.E., A.O.

Data collection: S. Shigeta, M.S., S. Suzuki, K.O.

Writing―original draft: S. Shigeta, M.S.

Writing―review and editing: S. Shigeta, M.S., S. Suzuki, K.O., K.T., T.E.

Supervision: H.K., K.T., M.M., D.A., T.E., A.O.

### Approval by Institutional Review Board (IRB)

C-CAT database analysis

#2021341G (The University of Tokyo)

#CDU2022-026N (C-CAT review board)

CYH33-G201 trial

#T4962 (National Cancer Center)

JGOG2052

#I021-002 (Niigata University)

JGOG3029

#I022-002 (Niigata University)
